# Lack of association between a functional variant of the BRCA-1 related associated protein (*BRAP*) gene and ischemic stroke

**DOI:** 10.1186/1471-2350-14-17

**Published:** 2013-01-28

**Authors:** Yi-Chu Liao, Hsiu-Fen Lin, Yuh-Cherng Guo, Chung-Hung Chen, Zhi-Zhang Huang, Suh-Hang Hank Juo, Ruey-Tay Lin

**Affiliations:** 1 Section of Neurology, Taichung Veterans General Hospital, No. 160, Sec 3, Chung-Kang Rd, Taichung, 40705, Taiwan; 2 Department of Medical Genetics, College of Medicine, Kaohsiung Medical University, No. 100, TzYou First Road, Kaohsiung, 80708, Taiwan; 3 Department of Neurology, College of Medicine, Kaohsiung Medical University, No. 100, TzYou First Road, Kaohsiung, 80708, Taiwan; 4 Department of Neurology, National Yang-Ming University School of Medicine, No.155, Sec.2, Linong Street, Taipei, 11221, Taiwan; 5 Department of Neurology, Kaohsiung Medical University Hospital, No. 100, TzYou First Road, Kaohsiung, 80708, Taiwan; 6 Department of Medical Research, Kaohsiung Medical University Hospital, No. 100, TzYou First Road, Kaohsiung, 80708, Taiwan; 7 Department of Neurology, Changhua Christian Hospital, No. 135, Nanxiao St, Changhua, 500, Taiwan; 8 Department of Neurology, Yuan-Sheng Hospital, No.359, Juguang Rd, Changhua, 510, Taiwan

**Keywords:** *BRAP*, Polymorphism, Cerebral infarction, Myocardial infarction, Young stroke

## Abstract

**Background:**

Atherosclerosis shares common pathogenic features with myocardial infarction (MI) and ischemic stroke. BRCA-1 associated protein (*BRAP)*, a newly identified risk gene for MI, aggravates the inflammatory response in atherosclerosis. The aim of this study was to test the association between the *BRAP* gene and stroke in a Taiwanese population.

**Methods:**

A total of 1,074 stroke patients and 1,936 controls were genotyped for the functional SNP rs11066001. In our previous studies, the rare allele of this SNP has been repeatedly shown to exert a recessive effect. Therefore, in the current study, we tested for the same recessive model. First, the genotype distributions between all the controls and all the stroke cases were compared. Then to reduce heterogeneity, we explored several population subsets by selecting young stroke subjects (using 45 years of age as the cutoff point), age- and sex-comparable controls, plaque-free controls, and stroke subtypes.

**Results:**

We did not find any significant association for the entire data set (OR = 0.94, p = 0.74) or for the subset analyses using age- and sex-comparable controls (p = 0.70) and plaque-free controls (p = 0.91). Analyses of the four stroke subtypes also failed to show any significant associations (p = 0.42 – 0.98). For both young and old subjects, the GG genotype of rs11066001 was similar in the stroke cases and unmatched controls (8.1% vs. 9.4% in young subjects and 8.0% vs. 7.8% in old subjects). Comparing stroke cases with plaque-free controls also failed to find any significant association.

**Conclusions:**

The *BRAP* polymorphism may not play an important role in ischemic stroke in the studied population.

## Background

Stroke is a multifactorial disease attributable to both genetic and environmental factors. Twin and family studies suggest that genetic background plays a substantial role in stroke susceptibility [1,2]. A family history of stroke is associated with a higher risk for stroke with an odds ratio (OR) of 1.3 [1]. A family history of myocardial infarction (MI) is also associated with an increased risk for stroke, especially for large artery stroke [3]. These findings imply that these two diseases may have certain genetic or environmental risk factors in common. For example, the angiotensin converting enzyme (*ACE*) and the methyl-tetrahydrofolate reductase (*MTHFR*) genes confer risks for both stroke and MI [4-6]. The 9p21 locus was first found to be associated with MI and later, a meta-analysis of genome-wide association studies found that the same locus was related with large artery stroke [7].

Recently, the BRCA-1 associated protein (*BRAP*) gene was identified as a susceptible gene for MI [8]. The rare GG homozygote of the single nucleotide polymorphism (SNP) rs11066001 in the *BRAP* gene was found to be associated with a 1.31- to 1.47-fold risk for MI in Chinese and Japanese populations [8]. SNP rs11066001 has also been shown to be related to coronary artery disease (CAD) [9-11], ankle-brachial index [12,13] and carotid atherosclerosis [14]. In vitro studies showed that the G allele of SNP rs11066001 had an elevated *BRAP* expression, which exaggerated the activity of nuclear factor-kappa B (NF-κB) [8]. NF-κB is considered to be the “master regulator” of inflammatory cascades. Our recent study demonstrated that *BRAP* could enhance NF-κB nuclear translocation and subsequently initiated the transcription of inflammatory cytokines [14]. Because atherosclerosis can be caused by a chronic inflammatory condition, *BRAP* might play a role in the occurrence of ischemic stroke.

The heterogeneous entity of stroke poses challenges in identifying its genetic determinants. Focusing on individual stroke subtypes or studying intermediate phenotypes might help to reduce the complexity of the problem. Twin studies showed that the heritability of stroke was different across stroke subtypes and cardio-embolic stroke was found to be the least heritable [2,15]. Besides, the genetic influence on stroke susceptibility may decrease with increasing age [15]. Jerrard-Dunne and coworkers found that a positive family history of stroke carried an OR of 4.46 for having a stroke by the age of 55 years, an OR of 2.34 by 65 years, and an OR of 1.88 by 75 years [16]. This data implied that studying the role of a candidate gene in young stroke patients may provide a better chance of elucidating the genetic effect of the gene. The aim of the present study was to investigate whether *BRAP* confers a risk for stroke in a Taiwanese population. Polymorphism rs11066001 was selected because it is a functional locus that affects the transcription of *BRAP*[14], and rs11066001 has also been shown to have the strongest association with MI risk [8]. We performed subgroup analysis by stroke subtype and by stroke onset age. To our knowledge, this is the first study to investigate the effect of *BRAP* on the risk for stroke.

## Methods

### Study subjects

A total of 1,074 patients with ischemic stroke were enrolled from the Kaohsiung Medical University affiliated Hospitals and the Taichung Veterans General Hospital in Taiwan. All stroke patients were examined using brain computed tomography or magnetic resonance imaging to confirm the diagnosis of ischemic stroke [17]. Patients with hemorrhagic stroke, transient ischemic attack, cerebral vein thrombosis, or past histories of MI were excluded. The stroke subtypes were classified using the Trial of ORG 10172 in Acute Stroke Treatment (TOAST) [18]. For the control group, 1,936 stroke- and MI-free volunteers were recruited at the Kaohsiung Medical University affiliated Hospitals through an advertisement soliciting volunteers. All of the study participants were Han Chinese residing in Taiwan.

For both the stroke cases and control subjects, socio-demographic information and their medical history of hypertension, diabetes, hyperlipidemia, stroke, MI, and cigarette smoking were obtained. Body height and weight were measured for the calculation of body mass index (BMI). For the control subjects, blood pressure was measured using a calibrated standard aneroid sphygmomanometer (Omron; Vernon Hills, Illinois) after sitting for at least 5 min and the average value from two measurements was used. For the case subjects, blood pressure was measured daily during hospitalization and the mean of two measurements was used. Subjects were deemed to have hypertension if the systolic blood pressure was ≥140 mm Hg or diastolic pressure was ≥90 mm Hg, or if they were taking anti-hypertensive medications. Subjects were regarded as having diabetes if the fasting blood glucose was ≥126 mg/dl, or if they were taking hypoglycemic medications. Hyperlipidemia was defined by total cholesterol (TC) serum levels ≥ 200 mg/dl. Overnight fasting venous blood was collected for biochemical analyses and extraction of genomic DNA. Serum levels of fasting blood sugar, TC, high-density lipoprotein (HDL)-cholesterol, and triglyceride were determined using standardized enzymatic procedures (Boehringer Mannheim, Germany). The investigation conformed to the principles outlined in the Declaration of Helsinki. All study protocols were approved by local Institutional Review Boards (KMUH-IRB-950055, KMUH-IRB-980194 and VGHTC-IRB-C09067), and written informed consent was obtained from each participant.

### Carotid plaque and plaque-free subjects

All control subjects received carotid ultrasonic examinations for plaque detection; however, carotid plaque data was available only for a limited number of stroke cases. The Philips HD 11 ultrasonography system equipped with a 7.5 Hz to 10 Hz transducer (Philips Medical Systems, Washington, USA) was used. Carotid plaque was defined as an area of focal protrusion into the lumen that was at least 50% greater than the surrounding wall thickness. Control subjects were divided into two groups: (1) plaque-free subjects having no detectable plaque in the common carotid artery (CCA), carotid bifurcation (Bif), and internal carotid artery (ICA) on both sides of the body; and (2) plaque-prone subjects having at least one plaque in any of the six segments described above.

### DNA extraction and genotyping

Genomic DNA was isolated from the whole blood using a Puregene kit according to the manufacturer’s protocols (Gentra, Research Triangle, NC). Genotyping of *BRAP* rs11066001 was conducted using the TaqMan genotyping assay (Applied Biosystems, Foster City, USA). Briefly, PCR primers and two allelic-specific probes were designed to detect the specific SNP target. The PCR reactions were performed in 96-well microplates with an ABI 7500 real-time PCR machine (Applied Biosystems). Allele discrimination was achieved by detecting fluorescence using the ABI 7500 System SDS software version 1.2.3 (Applied Biosystems). The genotyping success rates were 96.4% in the control samples and 97.0% in the case samples.

### Statistical analysis

The genotype distribution was tested for Hardy-Weinberg equilibrium (HWE) using the goodness-of-fit test. In our previous association studies based on a Chinese population residing in Taiwan [7,8,12,13], the rare G allele of rs11066001 was found to exert a recessive effect on risk of stroke. Therefore, we focused our analyses on the recessive model of inheritance (GG genotype vs. AA+AG genotypes). Multivariate regression analysis with adjustment of traditional risk factors (including age, sex, diabetes, hypertension, hypercholesterolemia, and smoking) was used to evaluate the genetic effect of the risk genotype. The statistical analysis was performed with SPSS statistical software (version 13.0). An additional analysis based on the generalized odds ratio (OR_G_) was also performed. The OR_G_ is a model free approach without the assumption of any inheritance mode that uses the complete genotype distribution to estimate the magnitude of association between disease status and genotype [19]. The OR_G_ and 95% confidence interval (CI) were calculated using ORGGASMA software (available at http://biomath.med.uth.gr). Because we tested for five phenotypes (overall stroke and four stroke subtypes), a p-value of <0.01 was considered as statistically significant.

Several different case–control studies were explored, first be using all the study subjects, and then by selecting subgroups of the stroke subjects, including young stroke subjects. In addition, several strategies were used to select controls, first including all the controls, then using age- and sex-comparable controls, and plaque-free controls. First, we compared the genotype distribution between all the cases and all the controls. Cases were then stratified by stroke subtypes according to the TOAST classification [18]. Each stroke subtype was compared to all the controls using multivariate regression analysis as described above.

Because rs11066001 was associated with subclinical carotid atherosclerosis in our previous study [14], we chose a subgroup of plaque-free controls to serve as the “super-normal controls”. Genotype distribution was compared between all the cases and the plaque-free controls, and between each stroke subtype and the plaque-free controls. Because the genetic effect might be more prominent in young stroke patients, we also stratified the participants into younger subjects (age ≤45 years) and older subjects (age >45 years).

In addition, we selected subsets of age- and sex-comparable controls to eliminate the concern of a diverse age-and sex-distribution between cases and controls. Because the age of the stroke subjects ranged from 20 to 89 years, control subjects <20 or >90 years of age were excluded. To reduce age differences between the control and case subjects, we used a table of random numbers to remove control subjects between 20 and 50 years of age; all the stroke cases were retained until the age effect was no longer significant between cases and controls. To minimize the sex effect, we repeated the above procedure for men and women separately. Because men accounted for approximately two thirds of stroke cases, we removed a greater proportion of female controls than male controls; in particular, we deleted three female controls to one female case but only one male control to one male case was removed. The random numbers tables were generated by the SPSS software. After removing 830 of the controls, the age and sex data was similar between the two groups. We then performed multivariate regression analysis to compare the genotype distribution between all the cases and the age- and sex-comparable control group with adjustment for cardiovascular risk factors (including diabetes, hypertension, hypercholesterolemia, and smoking).

## Results

The genotype distribution of SNP rs11066001 was in HWE. The demographic features of the study participants are shown in Table 1. According to the TOAST classification, 247 cases (23.0%) were large-artery atherosclerosis, 118 were cardio-embolic infarct (11.0%), 457 were small-vessel occlusion (42.6%) and 252 were either other determined or undetermined etiologies (23.5%). Among the 1,936 controls, 1,283 were plaque free. A subset of 1,106 controls was selected to reduce the diverse age- and sex-distribution between cases and controls. The genotype distributions between all the controls, the age- and sex-comparable controls, and the plaque-free controls were very similar (Table 2).

**Table 1:** Demographic data of the study participants

	**Cases**	**Controls (All = 1936)**		
**Mean ± SD or%**	**All stroke cases (N=1074)**	**Age- and sex-comparable (N= 1106)**	**Plaque-free (N = 1283)**	**Plaque-prone (N = 653)**
Age (yr)	63.1 ± 11.5	62.3 ± 8.9	52.9 ± 10.1*	59.6 ± 9.8*
Male	703 (65.5%)	715 (64.6%)	476 (37.1%)*	315 (48.2%)*
Hypertension	727 (76.3%)	475 (43.1%)*	337 (26.3%)*	275 (42.2%)*
Diabetes	445 (46.7%)	176 (16.0%)*	102 (8.0%)*	109 (16.7%)*
Hypercholesterolemia	469 (43.7%)	648 (58.6%)*	722 (56.3%)*	426 (65.3%)*
Current or former smoker	460 (43.0%)	266 (24.4%)*	209 (16.4%)*	148 (22.9%)*
Total Cholesterol (mmol/L)	4.9 ± 1.1	5.2 ± 1.0 *	5.2 ± 0.9 *	5.3 ± 1.1*
HDL- Cholesterol (mmol/L)	1.1 ± 0.3	1.4 ± 0.3*	1.5 ± 0.4*	1.4 ± 0.4*
Triglyceride (mmol/L)	1.8 ± 1.7	1.5 ± 0.9*	1.4 ± 0.9*	1.5 ± 0.9*
Fasting blood sugar (mmol/L)	7.5 ± 3.7	5.7 ± 1.5*	5.5 ± 1.2*	5.8 ± 1.7*
Body mass index (kg/m^2^)	25.4 ± 3.8	24.8 ± 3.3*	24.4 ± 3.6*	24.7 ± 3.3*

* p < 0.05 in comparison to stroke cases using Chi-squared test or Student’s *t* test.

**Table 2:** The association between BRAP rs11066001 and stroke

		**rs11066001 genotype**			Recessive effect* (OR, Adjusted p value)		
		**AA**	**AG**	**GG**	**age- and sex-comparable**	**Plaque-free**	**All**
Stroke	All stroke cases (N = 1074)	571 (54.8%)	388 (37.2%)	83 (8.0%)	1.08, p = 0.70	0.97, p = 0.91	0.94, p = 0.74
Stroke Subtypes							
Large artery atherosclerosis (N=247)		132 (54.8%)	93 (38.6%)	16 (6.6%)	1.01, p = 0.98	0.82, p = 0.64	0.81, p = 0.56
Cardio-embolism (N = 118)		62 (53.9%)	44 (38.3%)	9 (7.8%)	0.85, p = 0.71	0.70, p = 0.48	0.73, p = 0.50
Small vessels occlusion (N=457)		246 (55.5%)	162 (36.6%)	35 (7.9%)	1.24, p = 0.42	1.05, p = 0.86	1.01, p = 0.97
Other etiologies (N=252)		131 (53.9%)	89 (36.6%)	23 (9.5%)	1.26, p = 0.43	1.05, p = 0.87	1.09, p = 0.77
Controls	age- and sex-comparable controls (N = 1106)	529 (49.5%)	458 (42.9%)	81 (7.6%)	ref.	--	--
	Plaque-free controls (N = 1283)	619 (50.4%)	518 (42.2%)	90 (7.3%)	--	ref.	--
	All controls (N = 1936)	929 (49.8%)	787 (42.2%)	150 (8.0%)	--	--	ref.

Adjusted p value was obtained from logistic regression with adjustment for traditional risk factors (age, sex, diabetes, hypertension, hypercholesterolemia, and smoking).

We did not find any association between *BRAP* SNP rs11066001 and the group with all the stroke cases using any of the three control groups (the entire group, the age- and sex-comparable control group, and the plaque-free group) (Table 2). The frequencies of the AA, AG and GG genotypes were 54.8%, 37.2% and 8.0% in stroke patients and 49.8%, 42.2%, and 8.0% in the group with all the controls. Compared to the (AA + AG) group, the GG genotype group had no increased risk for stroke. The OR_G_ was 0.85 (95% CI = 0.74-0.97) from the ORGGASMA analysis for the comparison between the groups with all the stroke cases and all the controls (Additional file 1). However, the significance disappeared after adjusting for cardiovascular risk factors using multivariate regression analysis (OR = 0.94, p = 0.74). A similar analyses for the four stroke subtypes against the group with all the controls also failed to find any significant associations (p = 0.50 – 0.97, Table 2).

When the stroke patients were compared with the subset of age- and sex-comparable controls, the genotype distribution of rs11066001 was similar between the two groups. The frequencies of GG genotype were 8.0% in the group with all the stroke patients and 7.6% in the age- and sex-comparable control group (Table 2). Compared to the (AA + AG) genotype, the GG genotype did not increase the risk for overall stroke (OR = 1.08, p = 0.70). Analyses of the four stroke subtype groups also failed to show any significant associations (p = 0.42 – 0.98, Table 2). However, the level of the genetic effect of rs11066001 appeared to differ among the stroke subtypes. Compared to patients with AA or AG genotypes, patients with the GG genotype had the highest risk for small vessel occlusion (OR = 1.24), followed by large artery atherosclerosis (OR = 1.01), and the GG genotype did not increase the risk for cardio-embolic infarct (OR = 0.85), although none of these results reached significant levels.

Similar to the previous analyses, when we used plaque-free controls to evaluate the genetic effect of rs11066001 we failed to find any significant association between rs11066001 and stroke (OR = 0.97, p = 0.91, Table 2). The frequencies of the GG genotype were similar between the group with all cases and the plaque-free control group (8.0% vs. 7.3%, Table 2). The generalized odds ratio model also failed to find a significant result (OR_G_ = 0.88 (95% CI: 0.75-1.02), Additional file 1). Analyses of the groups with the four stroke subtypes yielded negative results (p = 0.48 – 0.87, Table 2).

To find out if the genetic effect might be more evident in young stroke patients, we stratified the subjects into under 45 years and over 45 years age groups. For both the younger and older subjects, the GG genotype of rs11066001 did not differ between stroke cases and the unmatched controls (8.1% vs. 9.4% in the younger subjects; 8.0% vs. 7.8% in the older subjects, Table 3). When the stroke cases were compared with the plaque-free controls, no significant association was found.

**Table 3:** Stratification analysis

**Subgroup**	**Younger subjects (age <= 45 y/o)**				**Older subjects (age > 45 y/o)**			
	**AA + AG**	**GG**	**OR, adjusted p value**		**AA + AG**	**GG**	**OR, adjusted p value**	
Stroke cases	68 (91.9%)	6 (8.1%)	OR = 0.73,	OR = 0.71,	891 (92.0%)	77 (8.0%)	OR = 0.94,	OR = 0.99,
			p = 0.60	p = 0.58			p = 0.74	p = 0.96
Unselected controls	269 (90.6%)	28 (9.4%)	ref.	--	1447 (92.2%)	122 (7.8%)	ref.	--
Plaque-free controls	237 (91.2%)	23 (8.8%)	--	ref.	900 (93.1%)	67 (6.9%)	--	ref.

Adjusted p value was obtained from logistic regression with adjustment for traditional risk factors (age, sex, diabetes, hypertension, hypercholesterolemia, and smoking).

## Discussion

In the present study, we investigated the possible association between the genotypes of *BRAP* SNP rs11066001 and ischemic stroke in a Taiwanese population. Although several strategies were used to categorize cases and controls into relevant subgroups, none of our analyses showed any evidence of a significant association between the SNP and stroke. According to our previous study [8], SNP rs11066001 was associated with an OR of 1.31 for MI in a Chinese population. Using the Genetic Power Calculator (http://pngu.mgh.harvard.edu/~purcell/gpc/) estimation [20], our sample size for all the cases and controls provided a power of 74% for an OR of 1.31 and 98% for an OR of 1.50 with an alpha level of 0.05. Therefore, we concluded that SNP rs11066001 in the *BRAP* gene was unlikely to play an important role in stroke susceptibility in the Chinese population.

Previously, we reported that *BRAP* can modulate NF-κB activity [14] which in turn initiates the transcription of downstream inflammatory genes. We also found that *BRAP* may influence the secretion of inflammatory cytokines, and the proliferation and migration of smooth muscle cells [14]. Inflammation is an important element in stroke pathophysiology. Previous studies have shown that elevated high-sensitive C-reactive protein (hs-CRP) levels are associated with a 1.27-fold risk for ischemic stroke [21]. Several inflammatory genes, like interleukin-6 (*IL-6*) [22,23], tumor-necrosis factor-alpha (*TNF-α*) [24,25] and matrix metalloproteinase-3 (*MMP-3*) [25,26], were also found to confer risks for stroke. The above evidence justifies our effort to test for an association between *BRAP* polymorphisms and ischemic stroke.

SNP rs11066001 in the *BRAP* gene has been shown to be significantly associated with MI/CAD [8,9,11], carotid plaques [14] and Ankle-Brachial index (ABI) [12,13]. These associations imply that its genetic effect might be more evident in large artery atherosclerosis. However, we acknowledge that the sample size used in this study might be underpowered to detect a modest genetic effect on this stroke subtype. For large artery stroke, our sample size only provides a power of 33% for an OR of 1.31 and 71% for an OR of 1.50 with an alpha level of 0.05. MI is the clinical outcome of systemic atherosclerosis, while stroke is attributed to a more heterogeneous pathogenesis [27]. Therefore, a discrepant genetic effect between MI and stroke is not uncommon [28,29]. For example, a meta-analysis for 11 MI risk genes identified from genome-wide association studies found that none of the 11 genes increased the risk for ischemic stroke [28].

The search for stroke susceptibility genes has been extremely challenging. Previous studies related to risk genes for stroke yielded inconsistent results. Two widely investigated genes, phosphodiesterase 4 D (*PDE4D*) and lipoxygenase-activating protein (*ALOX5AP*), have never been confirmed in meta-analyses of stroke [30,31]. The complexity of stroke entities is considered to be one of the major causes for this. Large-artery stroke shares pathogenesis and risk factors with systemic atherosclerosis, including CAD and peripheral artery disease (PAD) [27,32]. On the other hand, small-vessel occlusion is caused by leukoaraiosis secondary to hypertension. When we tried to analyze the stroke subtypes, we encountered the problem of lack of power. Therefore, whether or not *BRAP* SNP rs11066001 can influence a particular type of stroke needs further investigation.

Susceptibility genes may have a more prominent influence on early-onset of the disease when environmental and behavioral factors have not yet had sufficient time to modify the phenotype. Age-dependent effects have been reported for *PDE4D* genes [33], variants at chromosome 9p21 [34,35], *MTHFR* and apolipoprotein E(*APOE*) genes [36]. Although we only had 74 young stroke cases in our study population, the genotype distributions between the younger and older stroke cases (ages less than and greater than 45 years respectively) were essentially identical. Therefore, we propose that age is unlikely to modify the relationship between *BRAP* gene and stroke.

The associations between *BRAP* and vascular diseases were intriguing. *BRAP* polymorphisms have been related to metabolic syndrome in a Chinese population [37], MI/CAD in Japanese, Korean and Taiwanese populations [9-11], and ABI and carotid atherosclerosis in a Taiwanese population [12-14]. Only one report showed that a *BRAP* polymorphism was associated with metabolic syndrome in European American and African American populations [38]. It is unclear whether findings from the *BRAP* SNP studies can be generalized to non-Asian populations.

## Conclusions

In the present study, we failed to find any significant association between *BRAP* SNP rs11066001 and stroke in a Taiwanese population. Although our previous functional studies demonstrated a role of *BRAP* in the pathogenesis of atherosclerosis and previous genetic association studies supported the role of *BRAP* polymorphisms in systemic atherosclerosis (CAD and PAD), our current results suggest that *BRAP* does not play a critical role for stroke. However, further large-scale studies to explore the role of *BRAP* in stroke subtypes are needed.

## Competing interests

The authors declare that they have no competing interests.

## Authors’ contributions

YCL carried out the molecular genetic studies, participated in the statistical analyses and drafted the manuscript. HFL, YCG and CHC participated in subject enrollments and acquisition of clinical data of study subjects. ZZH participated in the molecular genetic studies and statistical analyses. SHHJ and RTL participated in forming the study concept, conceived the study and drafted the manuscript. All authors read and approved the final manuscript.

## Pre-publication history

The pre-publication history for this paper can be accessed here:

http://www.biomedcentral.com/1471-2350/14/17/prepub

## Supplementary Material

Additional file 1: Table S1 The association between BRAP rs11066001 and stroke. Click here for file
