# Supplementary material for: Lack of association between a functional variant of the BRCA-1 related associated protein (BRAP) gene and ischemic stroke
Source: BMC Med Genet. 2013 Jan 28;14:17. doi: 10.1186/1471-2350-14-17 (PMC3564782; doi:10.1186/1471-2350-14-17)
Supplement: Additional file 1: Table S1 — The association between BRAP rs11066001 and stroke. [file 1471-2350-14-17-S1.pdf]

Supplementary Table 1: The association between *BRAP* rs11066001 and stroke

		rs11066001 genotype			All		Plaque-free	
		AA	AG	GG	OR, Adj p value	OR_G	OR, Adj p value	OR_G
Stroke	All stroke cases<br>(N = 1074)	571<br>(54.8%)	388<br>(37.2%)	83<br>(8.0%)	R:0.94, p = 0.74<br>D:0.88, p = 0.22<br>A:0.91, p = 0.27	0.85(0.74-0.97)*	R:0.97, p = 0.91<br>D:0.91, p = 0.42<br>A:0.94, p = 0.49	0.88(0.75-1.02)
Stroke Subtypes								
	Large artery atherosclerosis<br>(N=247)	132<br>(54.8%)	93<br>(38.6%)	16<br>(6.6%)	R:0.81, p = 0.56<br>D:0.86, p = 0.40<br>A:0.88,p = 0.36	0.83 (0.65-1.06)	R: 0.82, p = 0.64<br>D: 0.88, p = 0.52<br>A: 0.89, p = 0.48	0.85 (0.66-1.11)
	Cardio-embolism<br>(N = 118)	62<br>(53.9%)	44<br>(38.3%)	9<br>(7.8%)	R: 0.73, p = 0.50<br>D: 0.73, p = 0.16<br>A: 0.77, p = 0.17	0.87 (0.61-1.24)	R: 0.70, p = 0.48<br>D: 0.69, p = 0.14<br>A: 0.74, p = 0.14	0.90 (0.63-1.29)
	Small vessels occlusion<br>(N=457)	246<br>(55.5%)	162<br>(36.6%)	35<br>(7.9%)	R: 1.01, p = 0.97<br>D: 0.94, p = 0.66<br>A: 0.97, p = 0.74	0.83 (0.68-1.00)	R: 1.05, p = 0.86<br>D: 1.00, p = 0.99<br>A: 1.01, p = 0.93	0.85 (0.70-1.05)
	Other etiologies<br>(N=252)	131<br>(53.9%)	89<br>(36.6%)	23<br>(9.5%)	R: 1.09, p = 0.77<br>D: 0.95, p = 0.76<br>A: 0.99, p = 0.91	0.90 (0.70 -1.15)	R: 1.05, p = 0.87<br>D: 0.95, p = 0.78<br>A: 0.98, p = 0.88	0.93 (0.72-1.20)
Controls	Plaque-free controls<br>(N = 1283)	619<br>(50.4%)	518<br>(42.2%)	90<br>(7.3%)	--	--	ref.	ref.
	All controls<br>(N = 1936)	929<br>(49.8%)	787<br>(42.2%)	150<br>(8.0%)	ref.	ref.	--	--

R: recessive, D: dominant, A: additive

Adjusted p value (Adj p) was obtained from logistic regression with adjustment for traditional risk factors (age, sex, diabetes, hypertension, hypercholesterolemia, and smoking). Generalized Odds ratio (OR_G) was calculated using the "ORGGASMA" software (available at http://biomath.med.uth.gr).

* $p < 0.05$
